# Laparoscopic Versus Open Rectopexy for Rectal Prolapse: A Randomized Controlled Trial

**DOI:** 10.7759/cureus.14175

**Published:** 2021-03-29

**Authors:** Kailash Chandra Mohapatra, Narendranath Swain, Srikant Patro, Ashok Kumar Sahoo, Ashish Kumar Sahoo, Ashish Kumar Mishra

**Affiliations:** 1 Surgery, S.C.B. Medical College and Hospital, Cuttack, IND; 2 Surgery, S.C.B. Medical College and Hospital, Cutack, IND; 3 Surgery, Jawaharlal Institute of Postgraduate Medical Education and Research, Puducherry, IND

**Keywords:** rectal prolapse, incontinence, constipation, laparoscopic rectopexy

## Abstract

Introduction

Most of the patients with rectal prolapse complain of fecal incontinence followed by constipation. Surgery is the only definitive treatment option for rectal prolapse. There are two approaches: either transanal/perineal or transabdominal. The abdominal procedures can be done in the open laparotomy method or laparoscopically. Suture rectopexy is a very old and popular method of treating rectal prolapse. Nowadays, rectopexy by laparoscopic approach is considered the gold standard treatment for rectal prolapse. The study has been conducted to compare both the procedures and their outcomes in terms of conditions associated with rectal prolapse.

Methods

All consecutive patients with full-thickness rectal prolapse who had attended the surgery outpatient department were included in the study. The patients had undergone either open suture rectopexy or laparoscopic rectopexy after randomization. Assessment of postoperative pain, mean days of hospital stay, constipation, and incontinence score along with operative time, recurrence within six months of follow-up, and time to resume bowel activity were done. The patients were followed up for 18 months at regular intervals.

Results

A total of 58 patients were included in the study: 27 in the open group and 31 in the laparoscopic group. The operative time was 102 minutes versus 129 minutes (p=0.0001) in the open and laparoscopic groups, respectively. The laparoscopic group had an earlier resumption of bowel activity (3.1 days vs. 1.4 days [p=0.0001]); fewer days of hospital stay (6.8 days vs. 2.5 days [p=0.0001]), less postoperative pain (mean visual analogue scale score for pain on postoperative day one 4.0 versus 3.1 [p=0.0035] and on postoperative day two 3.8 versus 2.2 [p=0.0001]). There was no significant difference in postoperative constipation score and incontinence score between the two groups.

Conclusion

Laparoscopic rectopexy results in lesser postoperative pain, lesser hospital stay, and better patient satisfaction than open rectopexy.

## Introduction

Rectal prolapse is defined as a protrusion of the full-thickness of the rectum through the anal canal [1]. Internal rectal prolapse, also known as rectal intussusception is the prolapse of the rectal wall without protrusion through the anus. If only the rectal or anal mucosa is protruded, it is called the mucosal prolapse, which should be distinguished from full-thickness rectal prolapse. The definite etiology is not completely revealed to date. One of the many hypotheses states that an intussusception of the rectum 6-8 cm from the anal verge is the preceding point by which prolapse originated [2]. The most common coexisting conditions associated with rectal prolapse are a redundant sigmoid colon, diastasis of the levator ani, a deep cul-de-sac, a patulous anal sphincter, the lack of rectal-sacral attachments, pelvic floor laxity, weak sphincter complex, deep Douglas’ pouch, pudendal neuropathy, and loose rectal fixation [3].

Anal incontinence, constipation, mucus discharge, hemorrhage are some of the symptoms of patients with rectal prolapse. Most of the patients complain of fecal incontinence followed by constipation [4]. Surgery is the only definitive treatment option for rectal prolapse. All the available surgical options are aimed to eliminate the prolapse, correct associated functional abnormalities of incontinence or constipation, and prevent de novo bowel dysfunction. All these results can be achieved either by fixation of the rectum to the sacrum and/or resection or plication of the redundant bowel. There are two ways of approach, which can be either transanal/perineal or transabdominal. The abdominal procedures can be done in the open laparotomy method or laparoscopically. Suture rectopexy is one of the very old and popularised methods of treatment for rectal prolapse. Conventionally, laparoscopic surgery is associated with less postoperative pain and hospital stay. Nowadays, rectopexy by laparoscopic approach is considered the gold standard treatment for rectal prolapse [5]. However, there is very limited data available comparing open suture rectopexy with the laparoscopic method. Hence, the study has been conducted to compare both the procedures and their outcomes in terms of conditions associated with rectal prolapse.

## Materials and methods

The study was conducted in the department of general surgery at a tertiary care hospital in East India from January 2018 to March 2020. The study was approved by the Ethics Committee of the institute and has been performed following the ethical standards laid down in an appropriate version of the Declaration of Helsinki (as revised in Brazil 2013).

All consecutive patients with full-thickness rectal prolapse who had attended the surgery outpatient department were included in the study. The details of the patients and the findings were recorded after obtaining informed and written consent. The patients had undergone either open suture rectopexy or laparoscopic rectopexy. Assessment of postoperative pain, mean days of hospital stay, constipation, and incontinence score along with operative time, recurrence within six months of follow-up, and time to resume bowel activity were done. The patients were followed up for 18 months at regular intervals.

Patients who were below the age of 18 years, those with comorbidities (American Society of Anesthesiologists [ASA]-3 or more) and associated malignancies were excluded from the study.

The study was designed as a prospective, open-labeled, parallel-arm randomized controlled trial. Block randomization was carried out using a computer program with randomly selected block sizes of four and six. Allocation concealment was ensured by the serially numbered opaque sealed envelope (SNOSE) technique.

All the patients who fulfilled the inclusion criteria recorded their demographic profile (age, sex, weight, BMI). Patients were randomized into two groups using the SNOSE technique. One group underwent open rectopexy while the other group underwent laparoscopic rectopexy. All the patients were operated on by an experienced single surgeon.

Open technique

Under general anesthesia, a lower midline abdominal incision was given. The rectum was mobilized from the sacral hollow up to the tip of the coccyx taking care of the nervi erigentes, ureter, and superior rectal vessels. Then the mobilized rectum was fixed to the sacral promontory with 1-0 polypropylene suture. The abdomen was closed in a conventional manner.

Laparoscopic technique

Pneumoperitoneum was created using the Veress needle method after giving general anesthesia. One 10 mm umbilical incision was given for the telescope and two 5 mm incisions were given in the right iliac fossa and left iliac fossa (working port). The rectum was mobilized from the sacral hollow as mentioned in the open technique, drawn upwards, and fixed to the sacral promontory with the help of 1-0 polypropylene. The port sites were closed in a conventional manner.

Various outcomes between the two groups were compared. Primary outcomes were postoperative pain (visual analog scale), mean days of hospital stay, constipation (preoperative and postoperative according to visual analog scale), and incontinence score. Secondary outcomes were operative time, recurrence within six months of follow up and time to resume bowel activity.

All the patients were followed up at two weeks, one month, three months, six months, 12 months, and 18 months postoperatively.

Statistical analysis

Statistical analysis was performed using IBM SPSS Version 21.0 (IBM Corp, Armonk, NY, USA). Parametric numerical data were reported as mean ± standard deviation for continuous variables; nonparametric numerical data were represented as median (range). Student’s t‑test was used to compare numerical variables; Chi‑square test was used to compare qualitative data, and p<0.05 was considered statistically significant.

## Results

**Figure 1:**
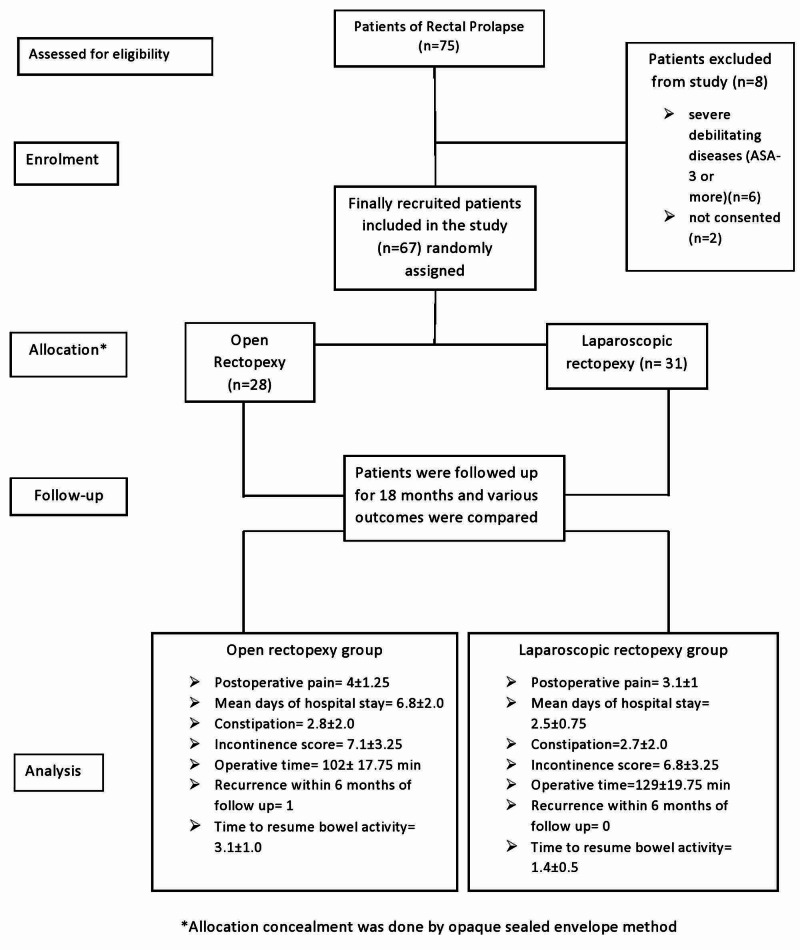
The overall scheme as per CONSORT flowchart CONSORT: CONsolidated Standards Of Reporting Trials; ASA: American Society of Anesthesiologists

In the present study, out of 75 patients with rectal prolapse, 67 were recruited for surgery (Figure 1).

Among the excluded patients, six were having severe debilitating diseases (ASA-3 or more), and the remaining two patients did not give consent to take part in the study. Twenty-eight patients were allocated to undergo open abdominal suture rectopexy and 31 patients had undergone laparoscopic suture rectopexy. One patient in the open group was excluded from the study due to his sudden demise in a car accident.

In the open group, 15 (55%) were male and 12 (45%) were female while 18 (58%) were male and 13 (42%) were female in the laparoscopic group. The mean age in the open group was 39.8 years and that of the laparoscopic group was 40.7 years. The mean weight was 70.2 kg and 69.8 kg in the open group and laparoscopic group, respectively. The mean BMI in the open and laparoscopic groups were 24.1 and 23.4, respectively (Table 1).

**Table 1:** Demographic profile of patients ^a^p-value (Chi-square test)

	Open group	Laparoscopic group	p-value^a^
Number of Patients	27 (M=15 (55%), F=12 (45%))	31 (M=18 (58%), F=13 (42%))	
Age (years)	39.8±10 (18-58)	40.7±10.5 (19-61)	0.740
Weight (kg)	70.2±13.5 (42-96)	69.8±12.75 (48-99)	0.908
BMI (kg/m^2^)	24.1±2.37 (18.8-28.3)	23.4±2.10 (19.3-27.7)	0.238

The mean operative time in the open group was 102 minutes (range 56-127 minutes) and 129 minutes (range 89-168 minutes) in the laparoscopic group (p=0.0001). The mean days to resume bowel activity was 3.1 days (range 1-5 days) and 1.4 days (range 1-3 days) in the open and laparoscopic group respectively (p=0.0001). The mean days of hospital stay were 6.8 days in the open group and 2.5 days in the laparoscopic group (p=0.0001). The postoperative pain in the laparoscopic group was lesser than in the open group (mean VAS of 4.0 in the open group as compared to 3.1 in the laparoscopic group on the first postoperative day [p=0.0035] and 3.8 compared to 2.2 on the second postoperative day (p=0.0001) in the open and laparoscopic groups, respectively) (Table 2).

**Table 2:** Outcomes of the study ^a^p-value (Chi-square test)

	Open group		Laparoscopic group		p-value^a^	
Operative Time (min)	102±17.75 (56-127)		129±19.75 (89-168)		0.0001	
Days to resume bowel activity	3.1±1.0 (1-5)		1.4±0.5 (1-3)		0.0001	
Days of hospital stay	6.8±2.0 (4-12)		2.5±0.75 (2-5)		0.0001	
Post-op VAS score for pain	Day 1	Day 2	Day 1	Day 2	Day 1	Day 2
	4±1.25 (3-8)	3.8±1.25 (3-8)	3.1±1 (2-6)	2.2±1.0 (0-4)	0.0035	0.0001
Incontinence score	Pre-op	Post-op	Pre-op	Post-op	Pre-op	Post-op
	7.1±3.25 (0-13)	2.0±2.25 (0-9)	6.8±3.25 (0-13)	1.9±2.0 (0-8)	0.727	0.858
Constipation score	Pre-op	Post-op	Pre-op	Post-op	Pre-op	Post-op
	2.8±2.0 (0-8)	2.6±1.75 (0-7)	2.7±2.0 (0-8)	2.8±2.0 (0-8)	0.85	0.68
Months of follow-up	13.1±4.25 (3-20)		12.8±4.0 (2-18)		0.783	
Recurrence	1		0		0.317	

The preoperative and postoperative constipation scores in the open group were 2.8 and 2.6 respectively whereas in the laparoscopic group were 2.7 and 2.8 respectively. The preoperative and postoperative incontinence score in the open group were 7.1 and 2.0 respectively and in the laparoscopic group were 6.8 and 1.9 respectively. The difference between the postoperative constipation score (VAS) (p=0.6889) and incontinence score (p=0.8584) was statistically insignificant between both groups. The mean months of follow-up were 13.1 months in the open group (3-20 months) and 12.8 months in (2-28 months) in the laparoscopic group. One patient in the open group had recurrence after six months of follow-up (p=0.317), which was statistically insignificant.

## Discussion

Although a lot of different surgical procedures have been proposed for rectal prolapse, not a single procedure has become the treatment of choice. Surgical management aims to restore the rectal physiology by correcting the prolapse and alleviating the symptoms such as incontinence and constipation with acceptable complications of surgery. Due to the comparative rate of recurrence and universally known benefits of minimally invasive surgery, laparoscopic procedures have been considered as the operation of choice for complete rectal prolapse [6]. Solomon et al. concluded that laparoscopic rectopexy is the preferred surgical option because of its nil long-term adverse outcomes [5]. The benefits of laparoscopic rectopexy over open rectopexy are all short-term. Laparoscopic rectopexy does not have any specific indications; it has the same indications as open rectopexy. The fact that prolapse repair procedures were an early indication within the sector of minimally invasive surgery reflects the relative simplicity of translating the open surgical techniques to the laparoscopic modality [7]. The open surgical treatment options for complete rectal prolapse are numerous [4, 8]. The common surgical steps among all these surgeries are rectal mobilization with fixation of the rectum to the sacrum either by the sutures or by a mesh. The outcome of the surgery can be enhanced by adding a resection and anastomosis of the recto-sigmoid [8]. A study from St. Mary’s hospital, London revealed that laparoscopic rectopexy can be done with good outcomes due to its shorter hospital span, diminished postoperative pain, and better cosmetic results [9]. Laparoscopic rectopexy involving any kind of mesh fixation increases the value of surgery, duration of the operation, and, therefore, the technical skills required to accomplish the operation in comparison to laparoscopic suture rectopexy. The addition of recto-sigmoid resection and anastomosis to laparoscopic rectopexy makes the procedure more technically demanding when practiced for all cases of complete prolapse of the rectum. There is a requirement to spot patients with complete prolapse of the rectum who are likely to profit from this procedure instead of recommending it for all cases.

Male patients were higher in number in the present study in the middle-aged group - that probably reflects the type of population covered by us, i.e., middle-aged group. The two most common symptoms associated with rectal prolapse are incontinence and constipation. Due to diminished rectal adaptation to distension in rectal prolapse, more than half of the patients have coexisting incontinence with rectal prolapse [10]. In the present study, the incontinence score had improved in both the groups similarly without any statistically significant difference. Constipation among both the groups also improved without any significant difference between them. Duration of surgery is one of the key parameters to be attributed to the advantages of an operation. In the present study, the duration of open rectopexy was 102± 17.75 (range 56-127) minutes while the time taken for laparoscopic rectopexy was 129±19.75 (range 89-168) minutes. The study done by Heah et al. reproduced that the average duration of laparoscopic rectopexy was 96 minutes (range 50-150 minutes) [11]. The longer duration in laparoscopy is understandable as it is technically challenging. The length of postoperative hospital stay is used as a yardstick for the patient's recovery and postoperative complications. In the present study, it was 6.8±2.0 (range 4-12) days in the open group while a patient stayed only for 2.5±0.75 (range 2-5) days in the laparoscopic group. Graf et al. showed a similar result regarding the duration of hospital stay after laparoscopic rectopexy [12]. Laparoscopic rectopexy is a relatively safe procedure with minimal morbidity and no mortality [11].

Bowel activity resumed earlier in the laparoscopic group as compared to the open group in the present study. It had taken 3.1±1.0 (range 1-5) days for the bowel activity to resume in the open group, while in less than half of that time the patients in the laparoscopic group started experiencing bowel activity. Milsom et al. demonstrated in their study an improved and early bowel activity but increased operative time in patients undergoing laparoscopic surgery for colorectal cancer [13]. 15-65% of patients with rectal prolapse had constipation as one of the associated symptoms [14]. In the present study, constipation in the postoperative period in both groups was comparable. Postoperative pain is as debilitating to the patient as to the surgeon. It is regarded as one of the important yardsticks in deciding the type of surgery for a particular disease. Stage et al. have shown that patients undergoing laparoscopic colorectal surgery had lesser postoperative pain than patients undergoing the open colorectal procedure [15]. In the present study, postoperative pain had been compared between the groups on day 1 and 2. The result showed the patients in the laparoscopic group had statistically significant lower pain as compared to the open group. Incidence of recurrence is regarded as one of the important parameters to evaluate the success of an operation.

Incidence of recurrence is one of the predominant criteria to measure the success of rectal prolapse surgery [16]. Graf et al. showed a recurrence of 0-3% after open rectopexy [12]. There is no significant difference in recurrence after laparoscopic rectopexy as well. Madiba et al. found a recurrence rate ranging from 0-10% after laparoscopic rectopexy [17]. In the present study, only one patient had a recurrence in the open group while none had any recurrence in the laparoscopic group. In toto, the present study had a recurrence rate of 1.5% (one in 67 patients). Thus, it becomes obvious that no matter the procedure, the recurrence rates do not show any significant difference.

The limitations of the present study are a smaller sample size, single-center study, and shorter period of follow-up. A larger trial may be done in the future to compare long-term outcomes between open and laparoscopic rectopexy.

## Conclusions

Postoperative pain and patient satisfaction are better in laparoscopic rectopexy than in open rectopexy. But the patients undergoing laparoscopic rectopexy have a longer operative time. Also, laparoscopic rectopexy needs technical expertise to perform.

The limitations of the present study are a smaller sample size, single-center study, and shorter period of follow-up. A larger trial may be done in the future to compare long-term outcomes between open and laparoscopic rectopexy.
